# Assisting PNA transport through cystic fibrosis human airway epithelia with biodegradable hybrid lipid-polymer nanoparticles

**DOI:** 10.1038/s41598-021-85549-z

**Published:** 2021-03-18

**Authors:** Marika Comegna, Gemma Conte, Andrea Patrizia Falanga, Maria Marzano, Gustavo Cernera, Antonella Miriam Di Lullo, Felice Amato, Nicola Borbone, Stefano D’Errico, Francesca Ungaro, Ivana d’Angelo, Giorgia Oliviero, Giuseppe Castaldo

**Affiliations:** 1 Department of Molecular Medicine and Medical Biotechnologies, University of Naples Federico II, 80131 Naples, Italy; 2 CEINGE-Biotecnologie Avanzate S.c.a.r.l., 80145 Naples, Italy; 3 Di.S.T.A.Bi.F., University of Campania Luigi Vanvitelli, 81100 Caserta, Italy; 4 Department of Pharmacy, University of Naples Federico II, 80131 Naples, Italy; 5 Institute of Crystallography, National Research Council, 70126 Bari, Italy; 6 ENT Section, Department of Neuroscience, Reproductive and Odontostomatological Sciences, University of Naples Federico II, 80131 Naples, Italy

**Keywords:** Biotechnology, Diseases, Molecular medicine, Chemistry

## Abstract

Cystic fibrosis (CF) is characterized by an airway obstruction caused by a thick mucus due to a malfunctioning Cystic Fibrosis Transmembrane Conductance Regulator (CFTR) protein. The sticky mucus restricts drugs in reaching target cells limiting the efficiency of treatments. The development of new approaches to enhance drug delivery to the lungs represents CF treatment's main challenge. In this work, we report the production and characterization of hybrid core–shell nanoparticles (hNPs) comprising a PLGA core and a dipalmitoylphosphatidylcholine (DPPC) shell engineered for inhalation. We loaded hNPs with a 7-mer peptide nucleic acid (PNA) previously considered for its ability to modulate the post-transcriptional regulation of the CFTR gene. We also investigated the in vitro release kinetics of hNPs and their efficacy in PNA delivery across the human epithelial airway barrier using an ex vivo model based on human primary nasal epithelial cells (HNEC) from CF patients. Confocal analyses and hNPs transport assay demonstrated the ability of hNPs to overcome the mucus barrier and release their PNA cargo within the cytoplasm, where it can exert its biological function.

## Introduction

Mutations in the Cystic Fibrosis Transmembrane Conductance Regulator (CFTR) gene, located on chromosome 7 and expressing for a chloride-conducting transmembrane channel protein in most epithelial cells, are responsible for the onset of the autosomal recessive genetic disease known as Cystic Fibrosis (CF). When both alleles of the CFTR gene include mutations, the cell produces an altered CFTR protein characterized by an absent or insufficient trafficking efficiency for water and chloride ions across the membrane of the affected cells^1^. This condition induces a medical phenotype comprising thick, sticky mucus in the airways, which clogs the bronchi and lungs, leading to repeated infections, with ultimate respiratory failure^2^.

In the past decades, the development of alternative therapies has become the main challenge for CF researchers. Among the different likely approaches, gene therapy provides a new opportunity to treat CF. We and others suggested miRNA-targeted strategies for the discovery of new treatments. miRNAs are small non-coding RNA strands, 20–24 nucleotides in length, that affect gene expression by mean of several mechanisms^3^. Most miRNAs downregulate the target genes by pairing the complementary 3’-untranslated region (UTR) of corresponding mRNAs. This event can induce the degradation of mRNA or hamper the translation of the encoded protein. It has been demonstrated that CF can be caused by the deregulation of specific miRNAs. Thus, our and other groups have explored the use of oligonucleotides (ONs) and their analogs to revert the activity of dysregulated miRNAs^4–6^. In particular, we have recently proven that the effects of miR-509-3p, previously reported to be one of the downregulating miRNAs of the CFTR gene^7^, can be reverted by using short peptide nucleic acids (PNAs) complementary to the first 14 bases^6^ or just to the seed region (only seven bases long)^8^ of this downregulating miRNA. PNAs are mimics of DNA and RNA with an uncharged backbone characterized by repetitions of N-(2-aminoethyl) glycine units^9^. The absence of phosphate groups in the PNA backbone reduces the electrostatic repulsion between PNA and its natural counterpart^10^. Thus, PNAs hybridize their Watson-Crick complementary RNA and DNA targets with higher affinity than corresponding unmodified ONs^11^. In addition, the low toxicity and the excellent stability in chemical and biological conditions render PNAs perfect candidates for the development of new gene therapies based on the anti-gene^12,13^, anti-sense^14^, and anti-miRNA^6,8^ strategies. However, the translation from feasibility studies to the production of PNA-based drug candidates has been slowed down by the low water solubility of PNAs longer than 12-14 bases and by the reduced capacity of such PNAs to cross the membranes of target cells^15^.

Mucopurulent masses, caused by the thick and sticky mucus, hold an important role in preventing drugs from targeting the site of infection. The low permeability of this barrier to molecules greatly limits the efficiency of treatments^2^. The use of nanocarriers, including nanoparticles (NPs) engineered for inhalation^16,17^, represents a promising alternative to control the release of drugs to their action site. Researchers are taking advantage of the opportunity to modulate the chemical-physical properties of inhalable NPs, thus improving drug loading/release, enhancing drug delivery across biological barriers, and tuning cell-NP interactions^18,19^. In this context, the development of NPs for pulmonary delivery of PNAs represents a fundamental challenge to overcome the biological barriers of CF sputum and bacterial biofilm^20^, which may exclude therapeutics from reaching the epithelium. Furthermore, the PNA cargo's slow release has the potential to prolong its residence time in the airways, enhancing its pharmacological effect and reducing the rate of drug appearance in the bloodstream (i.e., unspecific distribution to non-target tissues)^16,20^.

In this work, we attempted to engineer poly(lactide-*co*-glycolide) (PLGA)-based biodegradable and biocompatible NPs to achieve an efficient and prolonged local release of PNA within the mucus-covered CF bronchial epithelium. Exploiting an early investigated approach for nucleic acid delivery through the human epithelial airway barrier^21^, we focused our attention on hybrid core-shell NPs (hNPs) comprising a PLGA core and a dipalmitoylphosphatidylcholine (DPPC) shell. To this aim, we employed an ex vivo model of respiratory epithelium, based on human primary nasal epithelial cells (HNEC) from CF patients producing mucus and characterized by active cilia movement^22,23^. The HNEC cultures represent an excellent alternative model to human bronchial epithelial cell (HBE) cultures, maintaining most of the morphological and functional characteristics of HBE cells^24^.To follow the intracellular distribution and fate of PNA and hNPs, we conjugated the PNA to a fluorescein isothiocyanate (FITC) probe and the hNPs to Rhodamine B (Rhod). hNPs loaded with the FITC-labelled PNA (PNA*-hNPs) were produced and fully characterized, paying a particular attention to the in vitro release of PNA* from hNPs. We also assessed the ability of the PNA*-hNPs to penetrate the respiratory epithelium using the above-mentioned ex vivo model, based on HNEC.

## Results and discussion

In the last ten years, the encapsulation of different PNAs in PLGA NPs has been attempted to improve site-specific genome editing^25,26^, also to correct F508del CFTR in airway epithelium^27^. In this work, we try to harness PLGA NPs for pulmonary delivery of PNAs in CF. Conceiving this system for inhalation, PLGA NPs were surface-engineered with a layer of DPPC, achieving the so-called lipid-polymer hNPs, which recently demonstrated to be superior in nucleic acid delivery in the human airway epithelium^21^. According to our previous study, DPPC should be present on the surface of PNA*-hNPs, likely conferring them mucus inertia and, consequently, mucus-penetrating properties.

### Preparation and characterization of PNA*-hNPs

We designed the 7-bases long PNA* (Table 1) to be complementary to the seed region of miR-509-3p involved in the post-transcriptional regulation of the CFTR gene^8^. To improve solubility and complexation with lipofectamine, we installed the Gly-Ser(P)-Ser(P)-Gly peptide at the PNA* C-end. We also installed the FITC fluorescent probe at the N-end of PNA* for quantitation and cellular localization studies. PNA* was successfully synthesized as described in the “Methods” section by standard solid-phase synthesis and characterized by electrospray mass spectrometry (Fig. S1).

**Table 1:** PNA sequence used in this study.

Sample	Sequence	Structure
PNA* (C to N)	Gly-Ser(P)-Ser(P)-Gly-actaacc-(AEEA)2-FITC	

After the synthesis and purification of PNA*, PNA*-hNPs were prepared by emulsion/solvent diffusion, as described in the “Methods” section. The overall properties of PNA*-hNPs are reported in Table 2. PNA*-hNPs showed a hydrodynamic diameter lower than 200 nm and a negative z potential consistent with previously published measurements for siRNA-loaded hNPs^21^. The encapsulation efficiency of PNA* within the hNPs was higher than 90%.

**Table 2:** Size, polydispersity index (PDI), zeta potential, encapsulation efficiency, and PNA* loading in PNA*-hNPs.

	PNA*-hNPs
Size (nm ± SD)	174 ± 2.03
PDI (mean ± SD)	0.17 ± 0.012
Zeta potential (mV ± SD)	− 29.2 ± 1.58
Encapsulation efficiency (% ± SD)	94 ± 2.0
Actual load (nmol PNA/100 mg ± SD)	42.7 ± 0.899

**Figure 1:**
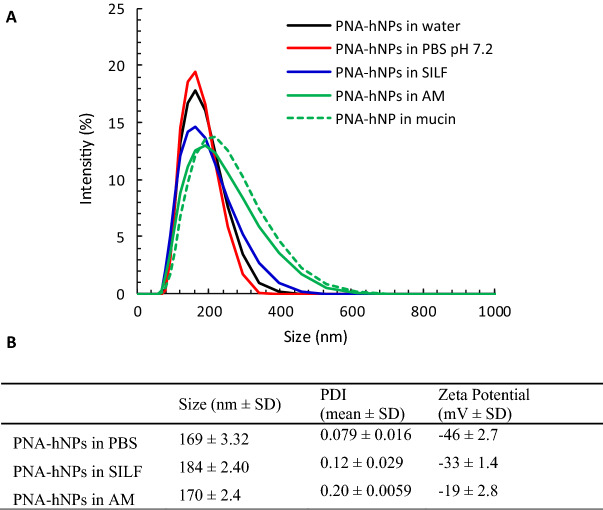
PNA*-hNPs properties in biologically relevant media. **(A)** Size distribution by intensity of PNA*-hNP dispersions in PBS pH 7.2, SILF and AM. The size distribution of PNA*-hNPs in water is reported for comparison. **(B)** Size, polydispersity index (PDI), zeta potential of PNA*-hNP dispersions in PBS pH 7.2, SILF and AM.

In order to evaluate the effect of the dispersing medium on particle properties, size, PDI and zeta potential of PNA*-hNPs were investigated also in biologically relevant media, that is PBS, simulated interstitial lung fluid (SILF), artificial CF mucus (AM) and mucin. As reported in Fig. 1, the size of the particles did not significantly change as a function of the dispersing medium. In particular, hNP behavior in mucin and AM suggested that hNPs are quite stable in the mucus environment. Indeed, only a slight increase in PDI was observed as compared to PBS. Likely as a consequence of the different ionic strength and composition of the tested media, slight changes were apparent in the absolute value of particle zeta potential, which is maintained negative in all the dispersing media (Fig. 1B).

### Release studies of PNA* from PNA*-hNPs

**Figure 2:**
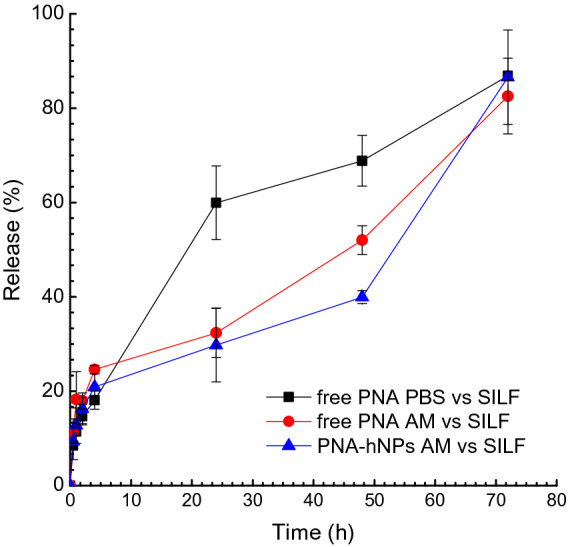
In vitro release profile of PNA* from PNA*-hNPs evaluated by membrane dialysis from artificial CF mucus (AM) to simulated interstitial lung fluid (SILF). Release profiles of free PNA* from AM to SILF and PBS to SILF are reported for comparison. Data are presented as mean value ± SD (n = 3).

We first evaluated the in vitro release kinetics of PNA* from PNA*-hNPs in sink conditions by simply suspending PNA*-hNPs in PBS pH 7.2 at 37 °C (Fig. S2). At scheduled time intervals, an aliquot of the release medium was withdrawn for quantification. As shown in Fig. S2, the developed PNA*-hNPs showed a biphasic release profile of PNA* with an initial burst of more than 60% of the encapsulated amount in 2 h followed by a sustained release up to 3 days. To better resemble in vivo conditions, in vitro release studies were also performed by dialysis from artificial CF mucus (AM) to simulated interstitial lung fluid (SILF) (Fig. 2). Free PNA* slowly diffused from PBS to SILF, with about 60% of the initial PNA amount added to the dialysis membrane available in SILF after 24 h of incubation at 37 °C. Diffusion of PNA* was retarded in the presence of AM, with a percentage of PNA* diffused in SILF at 24 h as low as 32.4 ± 5.2%. At 48 h, we found about 50% of PNA* in SILF. We observed a slight retardation effect for PNA*-hNPs, which released in SILF about 30% and 40% of the initial PNA* amount after 24 and 48 h, respectively. This slower kinetics can be likely due to the controlled release of PNA* from hNPs (60% of the initial amount released after 48 h, Fig. S2) and its subsequent diffusion from AM to SILF. After 72 h, we found a percentage as high as 85% of the entrapped PNA* in SILF. The mean hydrodynamic diameter of the present PNA*-hNPs, which is maintained lower than 300 nm after 72 h incubation in AM (Fig. S3), likely assisted the PNA diffusion through CF mucus^28^.

The stability of PNA*-hNPs in the release conditions was investigated by size analysis of particle dispersions after 72 h in AM and mucin at 37 °C. A slight increase in particle size was observed, which can be reasonably related to the complexity of the media compositions and their interreferences in the analysis. Nevertheless, the graph of particle size distribution suggested no particle aggregation both in AM and mucin (Fig. S3), confirming that hNPs are stable and muco-inert up to 72 h.

**Figure 3:**
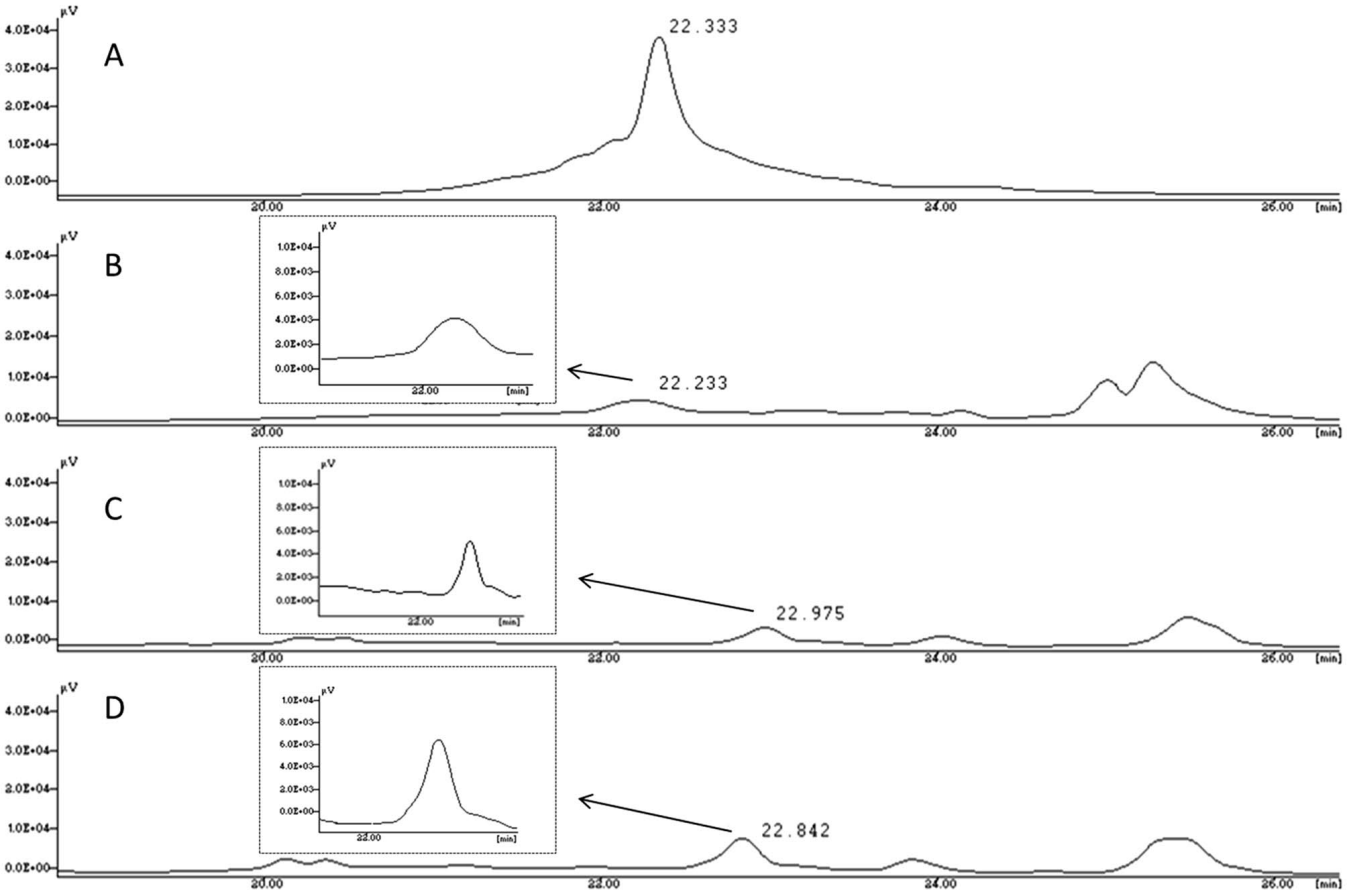
The RP-HPLC analyses of PNA* dissolved in H_2_O **(A)** is reported for comparison with the PNA* released from PNA*-hNPs AM vs. SILF after 24 **(B)**, 48 **(C)**, and 72 h **(D)**.

We also monitored the in vitro release of PNA* by RP-HPLC analysis. The retention time (Rt) of PNA* dissolved in H_2_O, around 22 min in Fig. 3 panel A, was compared with the Rt of PNA* released from PNA*-hNPs following the diffusion of the complex from AM to SILF at 24, 48, and 72 h (Fig. 3 panels B, C, and D, respectively). The analysis was performed by monitoring the UV absorbance of the PNA* bases at 260 nm. The chromatograms of PNA* released from PNA*-hNPs confirmed the presence of PNA* at all the investigated times.

Afterward, we carried out a preliminary quantitative analysis of PNA* released from hNPs using the ImageJ software tool. The results indicated that the area subtended by the peak of PNA* released from the complex at 72 h is approximately double in size than the area of the corresponding PNA* peak obtained at 48 h and roughly triple in size than the one calculated at 24 h (Table 3). These data were in agreement with the results shown in Fig. 2.

**Table 3:** Area (in pixel^2^) subtended by the peak corresponding to PNA* released from hNPs at 24, 48, and 72 h reported in Fig. 3, as calculated using the ImageJ software tool.

Sample	Area (pixel^2^)
PNA*-hNPs 24 h	4116
PNA*-hNPs 48 h	6399
PNA*-hNPs 72 h	11,314

### Transfection of the CF human airway epithelium model with PNA*

**Figure 4:**
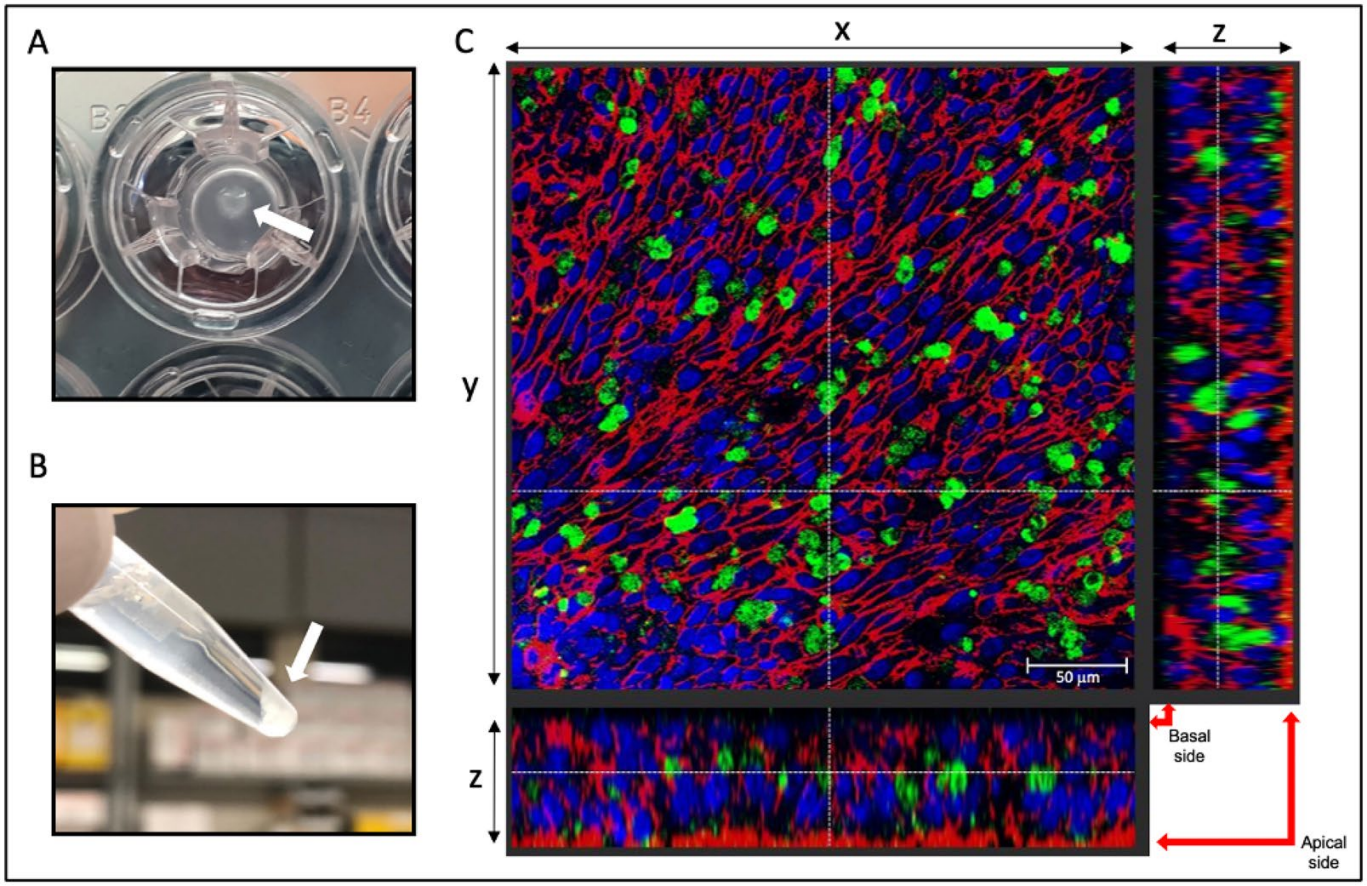
HNECs transfected with PNA*. **(A)** Apical view of ALI cultured HNECs with visible mucus, **(B)** pellet of collected mucus, **(C)** Z-stack confocal image of transfected HNECs. Green: PNA*, Blue: DAPI-stained nuclei, Red: phalloidin staining.

The effectiveness of PNAs to block miR-509-3p in A549 cells was previously investigated^5,8^. However, although of pulmonary origin, this cell line lacks many typical features of airway epithelia, which represents the target of the therapeutic use of our PNA-based system. To overcome this issue, we used a cellular ex vivo model based on primary human cells of nasal epithelium (HNEC). Indeed, the HNECs can form a pseudostratified epithelium when they grow on Transwell supports at Air–Liquid Interface (ALI), producing mucus and an active cilia movement. These features represent the two main obstacles to any aerosol treatment, with a more significant role of the mucus in CF patients. First, we evaluated the possibility of overcoming these obstacles by transfecting the HNECs with PNA* (Fig. 4A) to monitor the positive cells. The transfection was performed after 25 days of ALI culture when the mucus production (Fig. 4A,B) and ciliary beat were clearly visible. The confocal microscope analysis (Fig. 4C) showed that the PNA* was inside the epithelium cells (about 40% of cells). This analysis was performed using the ImageJ tool (data not shown).

### PNA*-hNPs transport through the CF human airway epithelium model

**Figure 5:**
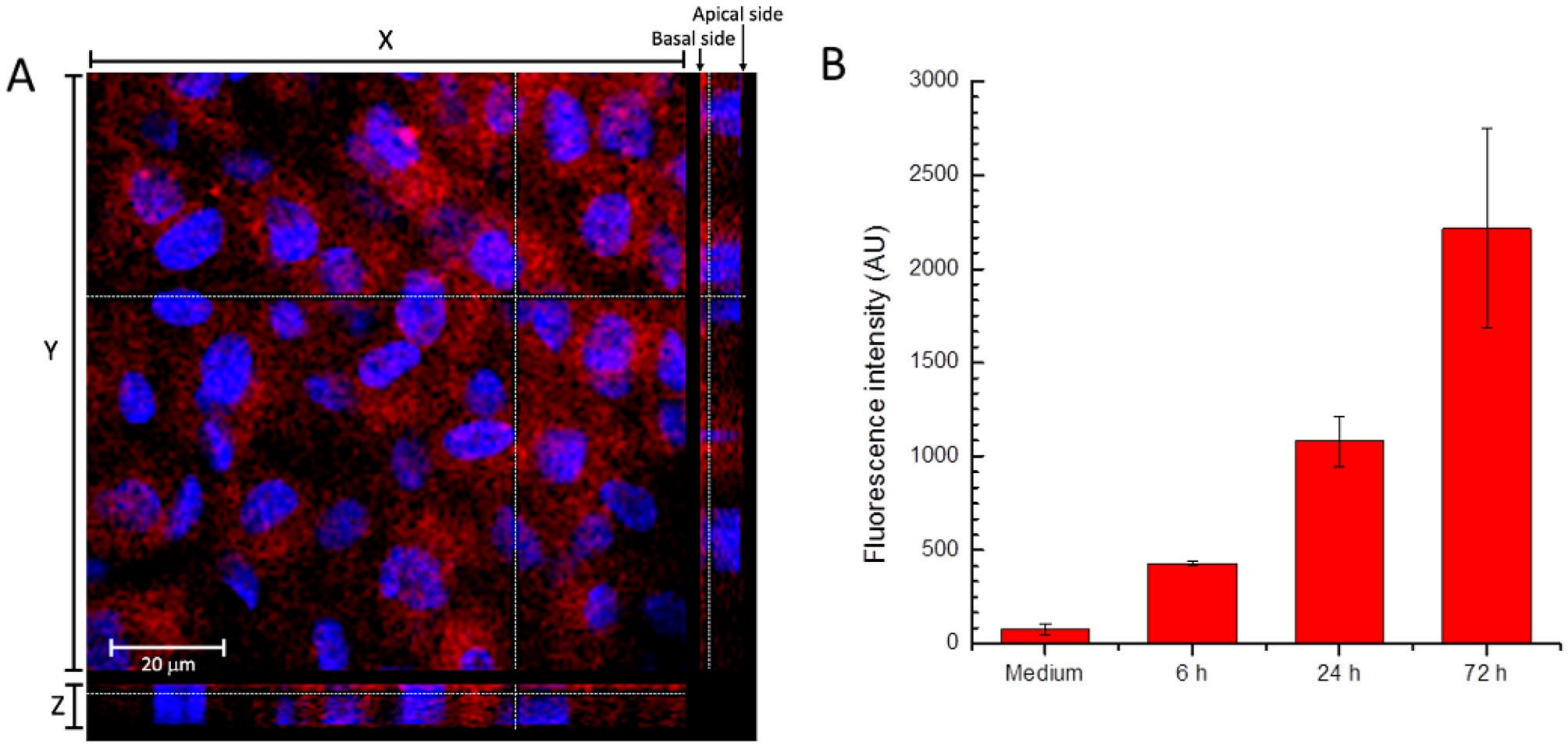
**(A)** Rhod-hNPs transport through the CF human airway epithelium model. Z-stack confocal image of HNECs treated with empty Rhod-hNPs for 6 h. **(B)** Fluorescence intensity of the basal medium of HNECs treated with 1 mg/mL of Rhod-hNPs at different time points.

Despite promising, the transfection of human airway epithelium with lipofectamine cannot be translated in vivo. Thus, we evaluated the ability of PNA*-hNPs to cross the ex vivo nasal epithelium. To this end, we first performed transport experiments through the polarized HNEC model of empty Rhodamine B-labeled hNPs (Rhod-hNPs). Notably, differently from what was observed in the HNECs transfected with PNA*, with only 40% of positive cells, the HNECs treated with Rhod-hNPs are 100% positive (Fig. 5A). The fluorescence intensity in the basal medium of HNECs after 6, 24, and 72 h had been monitored, demonstrating the progressive transport of the Rhod-hNPs through the epithelium (Fig. 5B).

**Figure 6:**
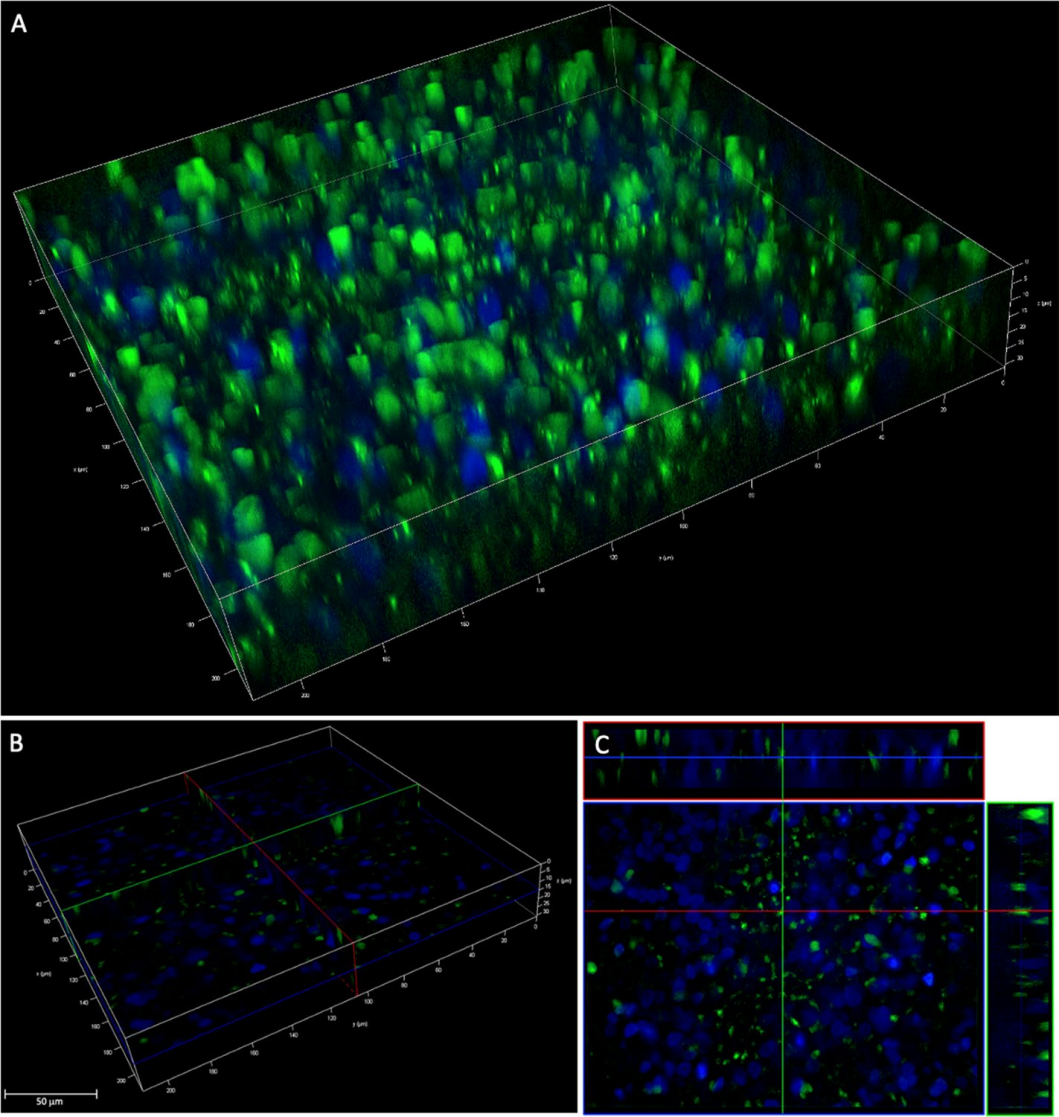
HNECs treated with hNPs loaded with PNA*. **(A)** 3D reconstruction of the Z-stack fluorescent images of HNECs treated for 6 h. **(B,C)** Relative sections of image **(A)**.

In light of these results, we assessed the ability of hNPs to assist PNA* transport through the lung epithelial barrier treating polarized HNECs grown with PNA*-hNPs. The results are reported in Fig. 6. Compared to the transfected cells reported in Fig. 4, even in this case, the cells were all positive for the FITC signal, confirming the ability of hNPs to deliver their load to 100% of the cells.

## Conclusion

In this work, we successfully developed core–shell hNPs comprising a PLGA core and a DPPC shell to assist the delivery of PNAs to the lungs. The characterization of the PNA*-hNPs revealed optimized properties, such as size, surface charge, high PNA* encapsulation efficiency, and provided a prolonged release of the entrapped PNA* in simulated lung fluids. The size and surface engineering of the developed hNPs resulted in the enhanced transport of the PNA in artificial CF mucus. The HPLC analysis showed that the synthetic mucus and the simulated interstitial lung fluid, mimicking in vivo conditions, do not influence the PNA* probe's integrity.

The produced NPs demonstrated, for the first time, their ability to penetrate the mucus and cellular barriers of the respiratory epithelium of an ex vivo model based on nasal epithelium cells. In particular, hNPs efficiently diffused across the cell membrane and were transported into the cytoplasm, where the target is located, releasing the PNA.

In conclusion, our results prompt towards the development of biodegradable nanodevices made up of FDA-approved materials for the delivery of PNAs. This approach holds the potential for an improvement in the treatment of lung deseases, hopefully increasing the concentration of active molecules in the site of action.

## Methods

### Synthesis of PNA*

The PNA oligomer (Table 1) was synthesized using the Fmoc solid-phase strategy according to the procedure used in our previous studies^5,6,8,13^. 50 mg of 4-methylbenzhydrylamine (MBHA) resin (0.5 mmol/g) was swelled in CH_2_Cl_2_ for 30 min and washed with dimethylformamide (DMF) (× 5). Then, the resin was treated with a solution of 20% piperidine solution in DMF for 10 min. After washings in DMF (× 5), the resin was treated with 5 equiv. Fmoc-Gly-OH dissolved in N-methyl-2-pyrrolidone (NMP) 0.2 M, 5 equiv. 1-[bis(dimethylamino)methylene]-1H-1,2,3-triazolo[4,5-b]pyridinium 3-oxid hexafluorophosphate (HATU) dissolved in DMF 0.2 M and 5 equiv. N,N-diisopropylethylamine (DIPEA)/7.5 equiv. lutidine for 40 min at RT. The coupling of two Fmoc-L-Ser[PO(OBzl)OH]-OH was achieved using the following conditions: 8 equiv. Fmoc-L-Ser[PO(OBzl)OH]-OH monomer dissolved in NMP 0.4 M, 8 equiv. HATU dissolved in DMF (0.4 M), and 8 equiv. DIPEA/12 equiv. lutidine for 15 h at RT. After the serine couplings, a glycine residue was attached on the N-terminal of the serine tract following the previously described coupling with the glycine monomer. PNA monomers were coupled using the following conditions: 5 equiv. monomer building block in NMP (0.2 M), 5 equiv. HATU dissolved in DMF (0.2 M) and 5 equiv. DIPEA/7.5 equiv. lutidine, 40 min at RT. After each coupling step, capping with Ac_2_O in the presence of pyridine was performed for 20 min at RT. Fmoc groups were removed by two treatments with a 5% solution of 1,8-Diazabicyclo[5,4,0]undec-7-ene (DBU) in DMF solution (5 min). After the assembly of the PNA tract, two 2-[2-(Fmoc-amino)ethoxy]ethoxyacetic acid linkers (Fmoc-AEEA-OH; Sigma) were coupled to the terminal amino group of PNA using the following conditions: 8 equiv. Fmoc-AEEA-OH dissolved in NMP (0.4 M), 8 equiv. HATU dissolved in DMF (0.4 M), and 8 equiv. DIPEA/12 equiv. lutidine. After the removal of the Fmoc group, 5 equiv. of fluorescein isothiocyanate (FITC) 0.2 M were dissolved in DMF/DIPEA (2.5:97.5 v/v), and the solution was added to the resin, which was gently shaken in the dark for 15 h. At the end of synthetic cycles, PNA* was cleaved from the solid support by treatment with trifluoroacetic acid (TFA)/anisole/ethanedithiol (9:1:1; v/v/v) for 4 h, and the product was precipitated with cold diethyl ether. Once recovered by centrifugation, the precipitate was washed twice with diethyl ether, dissolved in water, and finally lyophilized.

PNA* was obtained with a 48–50% overall yield (94–95% average yield for each coupling step as estimated by Fmoc spectrophotometric measurements after detachment of Fmoc groups from the resin). The crude sample was purified by semipreparative HPLC analyses, and purifications were carried out on a PU-2089 Plus pump (Jasco) equipped with a UV-2075 Plus UV detector (Jasco) using a 10 × 250 mm C-18 reverse-phase (Merck Millipore) column (particle size 5 µm) eluted with a linear gradient of CH_3_CN containing 0.1% (v/v) TFA in H_2_O (from 0 to 100% in 45 min, flow 1.2 mL/min). The collected fractions were lyophilized, and the final pure product was characterized by ESI-MS (positive mode): ESI-MS (*m/z*) calcd. for PNA* [M + 2H]^+2^ 1495.49, found 1495.4; calcd. for [M + 3H]^3+^ 997.33, found 997.3; calcd. for [M + 4H]^4+^ 748.24, found 748.2 (Fig. S1). The amount of PNA* sample dissolved in pure water was estimated by spectrofluorimetric analysis at λex 476 nm/λem 500–560 nm (GloMax Explorer, Promega). The response's linearity was evaluated at the concentration range of 0.01−5 nmol/mL (r^2^ ≥ 0.999).

### Production and characterization of PNA*-hNPs

PNA*-hNPs were prepared by an emulsion/solvent diffusion technique as previously reported^21^. Briefly, 100 µL of a water solution of PNA* (50 nmol/mL) were added to 1 mL of methylene chloride containing PLGA 502H (10 mg; 1% w/v)^28^ under vortex mixing (2400 rpm, Heidolph) and DPPC (0.5 mg/mL), achieving a water-in-oil emulsion (w/o). Just after mixing, the w/o emulsion was added to 12.5 mL of ethanol 96% (v/v) under moderate magnetic stirring, leading to the immediate formation of hNPs. The obtained formulation was diluted with 12.5 mL of Milli-Q water and maintained under stirring for 10 min. Afterward, the residual organic solvent was removed by rotary evaporation under vacuum at 30 °C (Heidolph VV 2000) to a hNP dispersion final volume of 5 mL. The obtained hNPs were isolated by centrifugation at 7000 rcf for 20 min at 4 °C (Hettich Zentrifugen) and dispersed in water to the desired concentration.

To evaluate PNA release kinetics and follow the intracellular distribution of PNA and hNPs, both the PNA and the hNPs were labeled with a fluorescent tag. The PNA was labeled using the FITC fluorophore as described above, while the hNPs were labeled with Rhodamine B by adding Rhod-labelled PLGA (PLGA-Rhod) (PolySciTech) in the organic phase at 10% w/w with respect to the total PLGA amount.

### Determination of size and zeta potential of hNPs in biological relevant media

The hydrodynamic diameter and the polydispersity index of hNPs were determined by dynamic light scattering (DLS), while the z potential was measured by electrophoretic light scattering (ELS) (Zetasizer Nano ZS, Malvern Instruments Ltd). The hydrodynamic diameter and the z potential were evaluated on a hNPs dispersion (1 mg/mL) in different media: water, PBS (120 mM NaCl, 2.7 mM KCl, 10 mM Na_2_HPO_4_) at pH 7.2, simulated interstitial lung fluid (SILF) and artificial CF mucus (AM). Briefly, SILF was prepared dissolving in water 0.095 g MgCl_2_, 6.019 g NaCl, 0.298 g KCl, 0.126 g Na_2_HPO_4_, 0.063 g Na_2_SO_4_, 0.368 g CaCl_2_ dihydrate, 0.574 g of sodium acetate, 2.604 g of NaHCO_3_, 0.097 g of sodium citrate dihydrate. The AM was prepared adding 250 μL of sterile egg yolk emulsion, 250 mg mucin, 0.295 mg diethylenetriamine pentaacetic acid (DTPA), 250 mg NaCl, 110 mg KCl, 1 mL of RPMI to 50 mL of water and the dispersion was stirred until a homogenous mixture was obtained. Due to the complexity of the medium composition, the AM was diluted in water (1:10 dilution) before hNPs dispersion in order to reduce the high interferences in the size and z potential analysis. To study in depth the interactions of hNPs with mucus component, the size and z potential analysis were additionally carried out on particle dispersion in a water suspension of mucin (0.08% w/v), one of the main components of mucus. Experiments were run in triplicate and results are reported as representative size distribution by intensity of hNP dispersions in different media and as mean value ± standard deviation (SD).

### PNA actual loading

To assess the actual loading of PNA* in the hNPs, we used both indirect and direct methods, whose results were compared for uniformity. In the indirect method, we detected the amount of non-encapsulated PNA*. Immediately after their production, the PNA*-hNPs were collected by centrifugation (7000 rcf for 20 min), and the supernatant was analyzed for PNA* content by spectrofluorimetric analysis, as described above. For the direct method, just after production, PNA*-hNPs were collected by centrifugation (7000 rcf for 20 min) and resuspended in water at the concentration of 1 mg hNPs/100 µL. 100 µL of the hNPs dispersion were then diluted with 900 µL of NaOH 0.5 N and incubated at RT for 2 h under magnetic stirring to achieve the polymer's complete degradation. The obtained solution was analyzed in triplicate by fluorescence spectroscopy, as described above, to quantify the amount of encapsulated PNA*. The two methods gave consistent results, reported in Table 2 as actual loading (nmol of encapsulated PNA* *per* mg of hNPs) and encapsulation efficiency (actual loading/theoretical loading × 100).

### PNA in vitro release kinetics from hNPs

The in vitro release kinetics of PNA* from PNA*-hNPs was first evaluated in PBS at pH 7.2. Release studies were performed upon dilution of PNA*-hNP dispersions in PBS to a final hNP concentration of 5 mg/mL. The obtained hNPs dispersions were incubated in a horizontal-shaking water bath at 37 °C (ShakeTemp SW 22, JULABO). At scheduled time intervals, the samples were centrifuged at 7000 rcf for 20 min to isolate hNPs. The release medium (1 mL) was withdrawn and reconstituted with the same amount of fresh PBS. The withdrawn medium was analyzed for PNA* content by spectrofluorimetric analysis as described above. Experiments were carried out in triplicate, and the results are expressed as the percentage of released PNA* ± SD. To better resemble in vivo conditions, the release of PNA* from hNPs was followed in vitro also by membrane dialysis from either PBS at pH 7.2 or AM to SILF according to the procedure previously described by De Stefano et al.^29^. The water dispersion of PNA*-hNPs (0.1 mL), corresponding to an amount of PNA* of 1 nmol, was added to 0.3 mL of donor medium (AM) and placed in a dialysis membrane bag (MWCO: 5000 Da, Spectra/Por). The dialysis membrane leads to the diffusion of the free PNA* and not the diffusion of the hNPs. The dialysis bag was placed into 3 mL of external medium and kept at 37 °C. At scheduled time intervals, 1 mL of external medium was withdrawn and analyzed for PNA* content by spectrofluorimetric analysis as described above. The medium was replaced by the same amount of fresh SILF. At the end of release kinetics, the amount of residual PNA* in the dialysis bag was assessed upon hNP degradation in NaOH 0.5 N, as described for PNA actual loading. The diffusion of free PNA* from AM to SILF and from PBS to SILF was evaluated in the same conditions as a control. Experiments were run in triplicate for each time point, and results are reported as the amount of PNA* diffused (%) in the time. The stability of hNPs in the release medium was assessed by size analysis of a PNA*-hNPs dispersion in AM (1 mg/mL) after 72 h of incubation at 37 °C. Due to the complexity of the AM composition, the sample was diluted in water (1:10 dilution) before analysis, in order to reduce the high interferences. The PNA*-hNPs dispersion in water and in mucin (water dispersion 0.08% w/v) were analyzed as control. Experiments were run in triplicate and results are reported as representative size distribution by intensity of PNA*-hNPs dispersion in AM.

### RP-HPLC analysis of PNA* in vitro release from hNPs

The in vitro release assay of PNA* dissolved in AM to SILF was assessed by reverse-phase (RP) HPLC following the absorbance of PNA bases at 260 nm. At scheduled time point, 500 µL of the stock solution of PNA*-hNPs from AM to SILF was withdrawn to perform the HPLC analysis, monitoring the UV signal of the PNA bases at 260 nm. The chromatogram of free PNA* in water was also analyzed in the same experimental conditions. The analyses were performed with a linear gradient of CH_3_CN containing 0.1% (v/v) TFA in H_2_O from 0 to 100% in 45 min, flow 1.2 mL/min at RT. Calculation of peak areas determined in pixel^2^ was performed using ImageJ software v1.52a^30^.

### hNP transport assay

Human nasal epithelial cells (HNEC) were treated with 1 mg/mL of empty Rhod-hNPs. The basal medium was recovered at different time points, 6, 24, and 72 h, and transferred to a clean 24-multiwell plate. The fluorescence intensity was measured by loading the 24-multiwell plate into a multi-plate reader (Enspire, PerkinElmer Italia). The excitation and emission wavelengths were set at 540 nm and 630 nm, respectively.

### Nasal epithelial cells brushing, culture, and hNPs treatments

HNECs were sampled by nasal brushing of both nostrils as reported by Di Lullo et al.^23^. Briefly, the nasal brushing was performed after nasal washings with a physiological saline solution to remove the mucus. Then, a soft sterile interdental brush (Paro Isola long, ProfiMed) with 2.5–3 mm bristles was used to scrap along the middle portion of the inferior turbinate, under direct vision, using a frontal light and without anesthesia. The brushes were transported to the cell culture lab in RPMI 1640 medium (supplemented with 1 mg/mL of primocin antibiotics—Invitrogen). The use of nasal epithelial cells from the human subjects was approved by the Ethical Committee of the University of Naples Federico II (protocol number 197/2015), in agreement with the Declaration of Helsinki. Written informed consent to collect and study the cells for scientific research purposes was obtained from the patients or, in the case of minors, from parents. Once in the lab, the cells were centrifuged at 350*g* for 10 min, resuspended in PneumaCult–EX Medium (STEMCELL Technologies), and seeded in a TPP T10 TC Flask/Tube 0.22 μm filter cap (Techno Plastic Products). Cell culture medium was changed every day. Then, about 33,000 cells were seeded on porous filters (0.33 cm^2^, Transwell, Corning) in PneumaCult–EX Medium until confluence. The PneumaCult–Ex Medium was replaced by PneumaCult-ALI Maintenance Medium (STEMCELL Technologies) for air–liquid interface (ALI) cultures. Treatments with hNPs (0.5 mg/mL), loaded or not with PNA*, were performed after 21 days of ALI culture when the pseudostratified epithelium was perfectly formed. Transfection of PNA* was performed using Attractene reagent (Qiagen).

### Fluorescent microscopy images acquisition

Cells were fixed in 10% neutral buffered formalin for 15 min, then rinsed in PBS and permeabilized with 0.1% triton x-100 in PBS for 10 min. After rinsing, the cells were blocked in 1% BSA solution for 30 min. then rinsed in PBS and then stained with phalloidin and Hoechst. The fluorescence images were acquired using a Leica SP5 confocal microscope, while the images of Fig. 4 were acquired using a THUNDER imager 3D cell cultures system (Leica).

## Supplementary Information

Supplementary Figures.
