# Supplementary material for: Assisting PNA transport through cystic fibrosis human airway epithelia with biodegradable hybrid lipid-polymer nanoparticles
Source: Sci Rep. 2021 Mar 18;11:6393. doi: 10.1038/s41598-021-85549-z (PMC7973768; doi:10.1038/s41598-021-85549-z)
Supplement: Supplementary file 1 — Supplementary Figures. [file 41598_2021_85549_MOESM1_ESM.pdf]

Supplementary material

Assisting PNA transport through cystic fibrosis human airway epithelia with biodegradable hybrid lipid-polymer nanoparticles

Marika Comegna[1,2†], Gemma Conte[3,†], Andrea Patrizia Falanga[4], Maria Marzano[5], Gustavo Cernera[1,2], Antonella Miriam Di Lullo[6], Felice Amato[1,2], Nicola Borbone[4], Stefano D'Errico[4], Francesca Ungaro[4], Ivana d'Angelo[3,*], Giorgia Oliviero[1,*] and Giuseppe Castaldo[1,2]

[1]University of Naples Federico II, Department of Molecular Medicine and Medical Biotechnologies, Naples, 80131, Italy
[2]CEINGE- Biotecnologie Avanzate S.c.a.r.l., Naples, 80145, Italy
[3]University of Campania Luigi Vanvitelli, Di.S.T.A.Bi.F., Caserta, 81100, Italy
[4]University of Naples Federico II, Department of Pharmacy, Naples, 80131, Italy
[5]National Research Council, Institute of Crystallography, 70126, Bari, Italy
[6]University of Naples Federico II, Department of Neuroscience, Reproductive and Odontostomatological Sciences-Ent Section, Naples, 80131, Italy
[*]corresponding authors: ivana.dangelo@unicampania.it; golivier@unina.it
[†]these authors contributed equally to this work

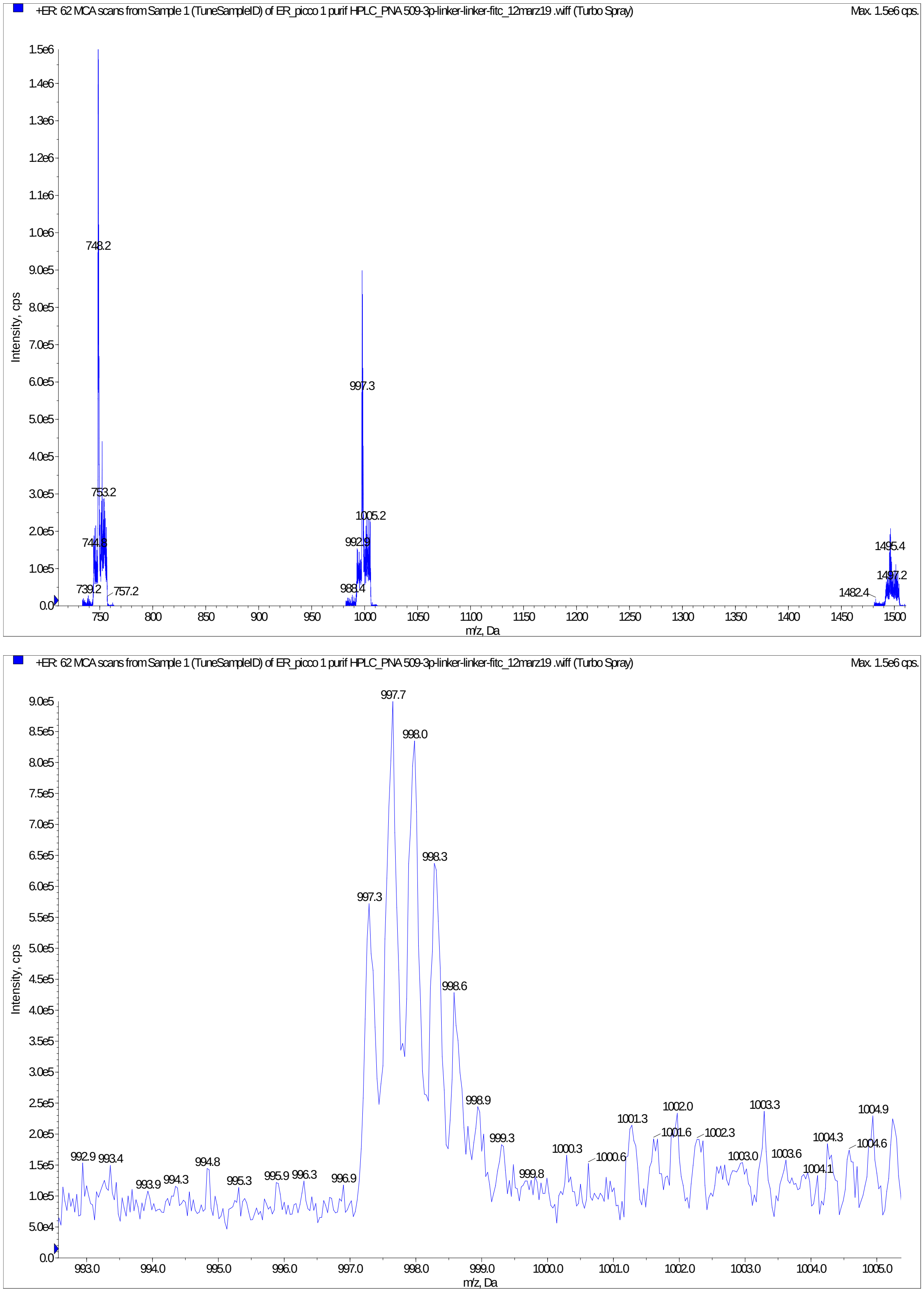

Figure S1: Expansions of the ESI-MS spectrum of PNA* recorded in the positive ion mode. Calcd. for PNA* [M + 2H]$^{+2}$ 1495.49, found 1495.4; calcd. for [M + 3H]$^{3+}$ 997.33, found 997.3; calcd. for [M + 4H]$^{4+}$ 748.24, found 748.2.

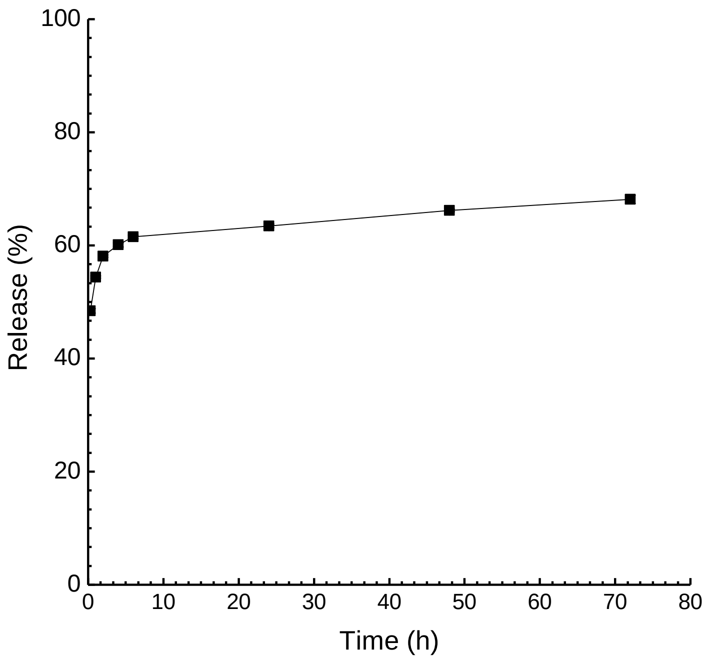

Figure S2: *In vitro* release kinetics of PNA* from PNA*-hNPs in PBS pH 7.2 at 37 °C.

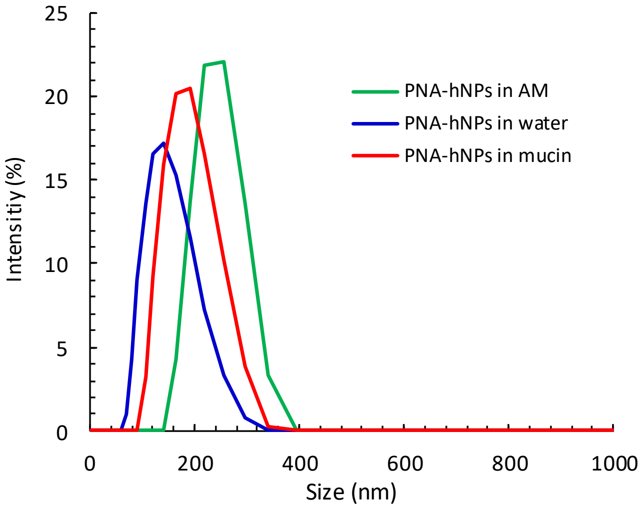

Figure S3: A: Size distribution by intensity of PNA*-hNP dispersions in mucin (water dispersion 0.08% w/v) and AM after 72 h at 37 °C. The size distribution of PNA*-hNPs in water is reported for comparison.
